# Graphene-Based Sensors for the Detection of Microorganisms in Food: A Review

**DOI:** 10.3390/bios13060579

**Published:** 2023-05-26

**Authors:** Jingrong Gao, Aniket Chakraborthy, Shan He, Song Yang, Nasrin Afsarimanesh, Anindya Nag, Shanggui Deng

**Affiliations:** 1 School of Food and Pharmacy, Zhejiang Ocean University, Zhoushan 316022, China; gaojingrong2116@sina.com (J.G.); dengshanggui@163.com (S.D.); 2 Faculty of Electrical and Computer Engineering, Technische Universität Dresden, 01062 Dresden, Germany; aniket.chakraborthy@tu-dresden.de; 3 Centre for Tactile Internet with Human-in-the-Loop (CeTI), Technische Universität Dresden, 01069 Dresden, Germany; 4 College of Engineering, IT & Environment, Charles Darwin University, Casuarina, NT 0810, Australia; 5 Institute for NanoScale Science and Technology, College of Science and Engineering, Flinders University, Bedford Park, SA 0810, Australia; 6 Yihai Food Technology Co., Ltd., Ma’anshan 243000, China; 7 School of Civil and Mechanical Engineering, Curtin University, Perth, WA 2605, Australia; nasrin.afsarimanesh@curtin.edu.au

**Keywords:** graphene, sensors, microorganisms, composites, *E. coli*

## Abstract

There is a constant need to maintain the quality of consumed food. In retrospect to the recent pandemic and other food-related problems, scientists have focused on the numbers of microorganisms that are present in different food items. As a result of changes in certain environmental factors such as temperature and humidity, there is a constant risk for the growth of harmful microorganisms, such as bacteria and fungi, in consumed food. This questions the edibility of the food items, and constant monitoring to avoid food poisoning-related diseases is required. Among the different nanomaterials used to develop sensors to detect microorganisms, graphene has been one of the primary materials due to its exceptional electromechanical properties. Graphene sensors are able to detect microorganisms in both a composite and non-composite manner, due to their excellent electrochemical characteristics such as their high aspect ratios, excellent charge transfer capacity and high electron mobility. The paper depicts the fabrication of some of these graphene-based sensors, and their utilization to detect bacteria, fungi and other microorganisms that are present in very small amounts in different food items. In addition to the classified manner of the graphene-based sensors, this paper also depicts some of the challenges that exist in current scenarios, and their possible remedies.

## 1. Introduction

The inclusion of sensors in different aspects of the lives of human beings has helped improve the quality of life to a great extent. In earlier times, after the initial use of complementary metal-oxide semiconductor (CMOS) circuits [1] for sensing purposes, semiconducting sensors [2,3] became popularized due to their wide operating range, high linearity and low input power. These sensors were mostly developed using the microelectromechanical systems (MEMS) technique [4,5] on single-crystal silicon substrates [6,7]. Although these sensors have served a great purpose for different industrial [8,9] and environmental [10,11] applications, some of the drawbacks associated with them [12] have led to the consideration of prototypes with alternative electromechanical properties. The second category of sensors were devised with materials having a certain degree of mechanical flexibility [13]. Some other advantages of flexible sensors include their low production costs, wider application spectra and faster responses towards stimuli [14,15]. These sensors are developed using a wide range of printing techniques [16,17,18]. Each of the fabrication techniques have allowed the processing of a wide range of polymers and nanomaterials to develop the resultant prototypes [19]. Among the nanomaterials, some of the common carbon-based allotropes [20,21], such as carbon nanotubes (CNTs) [22,23,24], graphene [25,26,27] and graphite [28,29,30], have been frequently used as a result of their excellent electrical conductivity, mechanical flexibility, biocompatible nature and easy customization with polymer matrixes. Out of these carbon allotropes, graphene has become a magic material, and has been used a great deal in its composite forms since 2007 [31]. It has been used to form single and multi-layered sensors for different kinds of biomedical [32,33], industrial [34,35] and environmental [36,37] applications. In addition to graphene and its various physicochemical forms, graphene oxide (GO) [38,39,40] and reduced graphene oxide (rGO) [41,42,43] have also been preferred due to their higher degree of dispersion in aqueous solutions. This paper shows the use of graphene-based sensors for the detection of different microorganisms, such as bacteria and fungi, in food items.

Among the different applications that the sensors have had a positive impact on, detecting food quality has been of great importance [44,45,46]. Food, with its different elemental constituents, has been the most important factor for human survival. The ever-increasing population and limited growth of food stocks have created a global food shortage over the years [47,48]. Among the food quantity generated per year, an unequal distribution makes it necessary for caretakers to preserve and main food quality. As the food is passed from the supermarket to the consumer over several days, its quality slowly degrades. Even though consumers are careful about food quality, there are a considerable number of cases when food is thrown away before it becomes inedible [49]. Thus, it is very important to know exactly until when food is still consumable without any microorganisms degrading its quality. Researchers are constantly working to create low-cost, robust sensing systems to determine and ubiquitously monitor the food quality, and decrease the overall wastage [50]. Among the different sensing materials that have been processed to form the prototypes for sensing food quality, graphene-based sensors have been significantly developed and deployed over the last decade. This paper highlights some of the significant examples of graphene sensors, where selective layers have been integrated to monitor the quality of food and detect different microorganisms present in them. Keeping in mind the recent pandemic and other food-borne diseases, the necessity of consuming good quality food has become of utmost importance. Food that is kept in unfavorable conditions can lead to the growth of bacteria and fungi, thus poisoning it. For example, if the food item is left in open or in humid conditions, there is a high chance for the growth of microorganisms in the food. Thus, it is the state of the art to know which types of microorganisms are present and can grow in food items over certain periods. Graphene sensors have been used to detect various targeted microorganisms in food constituents [51,52,53,54]. When the consumed food has a quantity of microorganisms that exceeds a certain limit, it becomes very dangerous. Due to these harmful microorganisms, different kinds of food-related diseases such as dysentery, diarrhea, salmonella and others exist [55,56]. Even though a lot of research has been carried out to detect the microorganisms present in food, a substantial review on using different graphene-based sensors for this purpose has not been conducted yet. This paper highlights the use of composite and non-composite graphene sensors to detect different kinds of microorganisms via the active layer of the prototypes. It was observed that all of these sensors are capable of performing as efficient sensors, with their respective performances differing with respect to the processed materials.

A lot of research has been conducted on devising methodologies to be used by graphene-based sensors to detect microorganisms in food items. Some of the common techniques include potentiometric sensing [57], electro-catalytic oxidation [58], fluorescent sensing [59], immunoassay [60], field-effect transistors [61] and impedance spectroscopy, which involve changes in the conductance [62], resistive [63] and capacitive values [64]. Each of these techniques have offered numerous advantages in the context of detecting certain microorganisms, such as bacteria and toxins, at very low levels. For example, potentiometric biosensors have provided certain edges such as their low costs, ease of use and their rapid responses. On the other hand, immunoassays have been able to carry out operations in a fast, simple and cost-effective manner. Non-invasive and facile manipulation has allowed researchers to use this technique effectively for planar biosensors. High sensitivities, specificity and accuracy are some of the reasons for using fluorescent sensing for this application. Each of these techniques have allowed graphene-based sensors to achieve quantitative detection of biomolecules in complex samples [65]. They have demonstrated significant performance in terms of their excellent sensitivity, stability and their wide detection range.

## 2. Graphene-Based Sensors

The succeeding examples describe the fabrication process of some types of graphene sensors, and their subsequent utilization for detection purposes of microorganisms in very small amounts. Each of these sensors are formed using different microfabrication techniques, chosen on the basis of the processed materials and final dimensions of the sensors. The processed materials chosen to integrate with graphene to form the prototypes included various polymers and other enzymes that were selective to the target bacteria and other microorganisms present in the food items.

Mohanty et al. [66] showcased research conducted for using GO to detect microorganisms present in food. These were composite sensors that involved the mixing of GO with polymer matrixes to form the active area of the prototypes. The presence of the polymers improved the mechanical integrity of the sensing area, and customized the effective electrical conductivity of the prototypes. The sensors were formed in a sandwiched structure, where an in situ polymerization process was carried out to develop the nano-biocomposites. The composites comprised GO sandwiched between poly (ethyl methacrylate) (PEMA)-co-starch and nano silver flakes. Some of the advantages of these sensors included their high barrier properties, biodegradable nature and antimicrobial behavior. The presence of GO and silver nanoparticles (Ag NPs) increased the oxygen barrier property of the PEMA-co-starch by around 40% and the degradation temperature with a residue of 24%. The Ag NPs were formed using ascorbic acid and starch as the reducing and stabilizing agents, respectively. The silver nitrate solutions were formed at four different molar concentrations before adding them to the ascorbic acid–starch solution. Then, the PEMA-co-starch was formed by initially optimizing the starch quality (5 wt. %) with respect to the PEMA, and then fixing it to optimize the PEMA for the composites. The next step was carried out by adding GO with a 2 wt. % to the resultant composites. Then, the samples were mixed with different amounts of Ag NPs to form the final composites. Four different types of bacteria were detected, with four different diameters of inhibition zones of 23 mm, 18 mm and 19 mm and 24 mm. The biodegradability of these nanocomposite-based sensors was found to be 4% after around 180 days.

Another study that highlighted the use of graphene-based sensors to detect microorganisms in food was shown in the research of Gouvêa et al. [67]. These composites included certain conductive and semiconducting nanomaterials such as rGO and zinc oxide (ZnO), respectively. The prototypes were synthesized using different techniques such as melt extrusion and reduction processes. The samples were mixed with glycerol-plasticized poly (3-hydroxybutyrate-co-3-hydroxy valerate) (PHBV) to form the hybrid sensors. After the initial preparation of GO, the rGO/ZnO composites were formed by mixing GO and zinc diacetate dihydrate under continuous stirring. While the pH of the samples was maintained at a specific value, the resulting mixture was heated to around 130 °C for six hours. Then, the samples were washed and dried in the oven at 60 °C for 48 h. Then, these samples were mixed with glycerol and PHBV at different weight ratios. The steps included homogenizing the samples in a conventional mixture, and then sealing them inside polyethylene bags at 4 °C for several days. The samples exhibited opposite effects when plasticizers and rGO/ZnO were added together, due to variations in the glass transition temperature. The prototypes were used for detection purposes by monitoring the bacterial activity during contact of the bacterial cells with the composite surfaces. The amount of ZnO that migrated into the surface was below the recommended level of zinc ions.

In a study conducted by Kotsilkov et al. [68], nanocomposites were formed using two different carbon-based allotropes, including PLA polymer-functionalized graphene nanoplatelets (GNP) and multiwalled carbon nanotubes (MWCNTs). The synthesis process was carried out using a 3D printing method, where the printed samples were hot pressed to form thin films having a thickness of around 30 microns. The PLA film samples were treated with ultra-strong migration at two different ethanol concentrations of 10 vol. % and 50 vol. %. Then, the samples were heated at 90 °C for four hours, and were stored for ten days at a fixed temperature of 40 °C. The microscopic images indicated the presence of smaller aggregates, below 500 nm, in 10 vol. % of ethanol. Simultaneously, platelets sizes of 100–1000 nm forming a few aggregates of 1–10 μm were mixed in 50 vol. % of ethanol concentrations. The scanning electron microscopic analysis also verified the presence of 10 μm GNP on the film surface at 10 vol. % ethanol concentrations. The thermal gravimetric analysis (TGA) and differential thermal gravimetry (DTG) study indicated the enhanced thermal stability of GNP-incorporated PLA over the pristine material. The transmission electron microscopic TGA study conducted at 850 °C on detecting residual ash confirmed the decrease in residual ash by 0.07%. It ensured the degradation of the maximum percentage of carbonaceous fillers.

**Figure 1:**
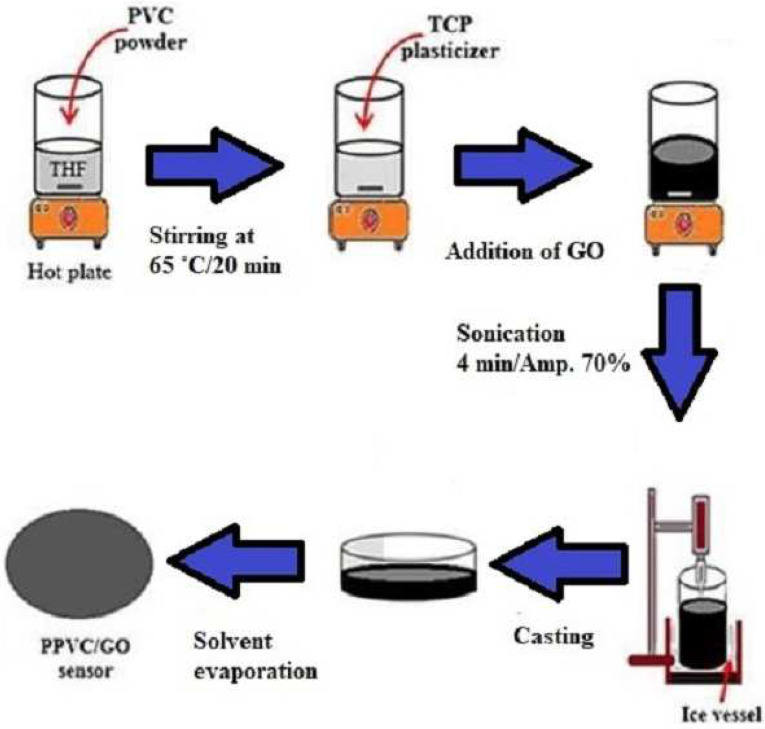
Schematic diagram of the fabrication process of the PPVC/GO sensors [69]. Ultrafast response humidity sensors based on polyvinyl chloride/graphene oxide nanocomposites for intelligent food packaging.

The research carried out by Moustafa et al. [69] can be related to food safety, as they used graphene to show the development of nanocomposite-based sensors for humidity-sensing applications. The research was carried out using plasticized polyvinyl chloride (PPVC) and tricresyl phosphate (TCP) as the processed materials. The nanocomposites were developed using three different proportions of GO nanoplatelets, including 1 wt. %, 3 wt. % and 6 wt. %. Figure 1 [69] shows the schematic diagram of the PPVC/GO sensor film fabrication process. An in situ chemical reaction technique was carried out, where the solutions were subjected to different processes, some of which included stirring at elevated temperatures, treatment in an ice atmosphere, sonication and drying. The sensors detected relative humidity over a wide range of 11–85% when tested for a frequency between 100 Hz and 100 kHz. This is necessary to detect changes in humidity where the food is being stored to monitor the growth of microorganisms in the food samples. The prototypes developed with a 3 wt. % of GO displayed the best performance as compared to the others. When the sensors were operated with an optimized frequency of 1 kHz, some of their achieved attributes showed high sensitivity and low hysteresis. The response and recovery times of the prototypes were around 4 s and 6 s, respectively. The quick and efficient response of these sensors is attributed to the absorption of water molecules due to the presence of the polar oxygen groups in GO and the oxygen atoms of TCP.

Barra et al. [70] showcased the development of chitosan-rGO-based flexible bio-nanocomposites for the detection of microorganisms in food items. These sensors were formed using an eco-friendly process, where a green methodology helped to develop prototypes with excellent electromechanical properties. Chitosan was formed by mixing it in distilled water and ultrasonication of the samples was conducted at 45 W for 20 min. After the rGO was hydrothermally reduced using caffeic acid, they were dispersed into chitosan at defined ratios to form nanocomposites. The loading amount of rGO was varied at five different ratios of 25%, 40%, 45%, 48% and 50%. The electrical conductivities of the samples were 0.7 S/m and 2.1 × 10^−5^ S/m in-plane and through-plane, respectively. The prototypes had additional advantages such as enhanced antioxidant activity and a mechanically reinforced chitosan matrix. Around 50% of rGO in the nanocomposites increased the tensile strength from 13 MPa to 27 MPa, and the Young’s Modulus from 0.47 GPa to 2.73 GPa. The resultant sensors displayed high tensile strength and decreased water solubility.

**Figure 2:**
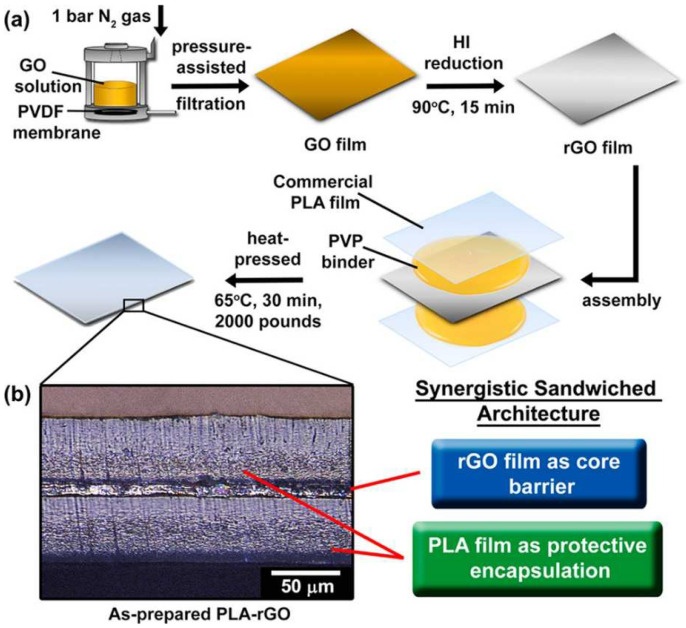
(**a**). Schematic diagram of the fabrication process of PLA/graphene composites. (**b**) Optical image highlighting the sandwiched structure of the developed samples [71]. Sandwich-architectured poly (lactic acid)–graphene composite food packaging films.

Another interesting study in this area is reference [71], where sandwiched nanocomposites were formed and employed to detect microorganisms in food items. Figure 2a,b [71] represent the fabrication process and optical image of the developed rGO/PLA nanocomposites-based prototype. The sandwiched structure was formed using rGO as the core barrier and commercial PLA films as the encapsulation layer. Large area GO and rGO films were formed using a pressure-assisted filtration process. The GO solutions were initially treated at a pressure of 1 bar in a nitrogen gas atmosphere. Then, free-standing rGO films were formed with a 12 × 12 cm^2^ dimension at 90 °C by treating the GO solutions with HI fumes. Finally, the PLA-graphene composite films were formed by cutting them into dimensions of 15 × 15 cm^2^, and then treating them with PVP solutions. The solution was prepared as a binder and the free-standing rGO films were delaminated from the composite films. Finally, the free-standing rGO films were placed between two PLA films and heat pressed at a temperature of 65 °C for half an hour. Two types of films, including PLA-rGO and PLA-GO films, were formed and deployed for sensing purposes. The sensors showed excellent permeability capabilities for water and air, as the water vapor permeability was reduced by up to 87.6% due to the synergy between the two processed materials of rGO and PLA. The air permeability of the prototypes also changed as the oxygen permeability was reduced, by a factor of two in both dry and humid conditions. The extensive and tortuous diffusion pathway of rGO allowed the nanocomposites to be formed with excellent processability.

Grande et al. [72] investigated the synthesis of GO-based nanocomposites and chitosan, and their potential for bacterial detection purposes. Some of the advantages of these sensors lay in their high mechanical integrity, excellent thermal stability and high antimicrobial activity. After the preparation of GO was carried out using a chemical process, the nanocomposites were formed by mixing chitosan and GO dispersions. The cross-linking between the GO and chitosan was created at a high temperature of 120 °C. The gel content assay revealed that incorporating GO increased the nanocomposite film’s gel percentage from 64% to approximately 89%. This may have been caused by the cross-linking interaction between the epoxy groups in the GO and the chitosan amine and hydroxyl groups. The FTIR analysis for the GO-based thin films showed that the wavenumber was observed at 1045, 1237, 1628 and 1739 cm^−1^. The presence of GO in 0.1 wt. % in the composite increased the initial tensile strength from 22.7 ± 1.2 to 6471.6 ± 1775.5 MPa. As the GO content was increased, there was a subsequent increase in the endothermal peak during thermal studies. It also increased the cross-linking degree between the film and water interaction. The nanocomposites were examined for antimicrobial properties using *E. Coli* K-12 MG 1655 and *B. subtillis* 102. The composite of 0.6 wt. % showed prime inactivation, which increased by 22.83% for *E. Coli* and 54.93% for *B. subtillis*.

Manikandan et al. [73] synthesized a polyhydroxy butyrate (PHB)-based nanocomposite for bacterial detection purposes. The fabrication of the nanocomposites was carried out using a solution casting method. While different solutions were mixed and stirred together, the films were evaporated and peeled off for further experiments. After the dissolution of PHB (0.1 g) in chloroform (10 mL), graphene nanoplatelets (Gr-NP) were prepared at various concentrations ranging from 0.3 to 1.3 wt. %, followed by dissolution (in chloroform) and sonication for 45 min. At an optimum concentration of 0.7 wt. %, the graphene nanoplatelet (Gr-NP)-incorporated PHB showed enhanced thermal stability and reduced cytotoxicity compared to the pristine PHB. The tensile stress and tensile stress values of the samples were 9 MPa and 12.2%, respectively. Regarding the biodegradation of the samples, they started degrading at the end of the fifth day and completely degraded at the end of the thirty days. The improved shelf life of the Gr-NP-based PHB makes it a suitable candidate for detecting bacteria and other microorganisms in food.

Naskar et al. [74] also reported the synthesis of nanocomposites-based anti-biofilms for bacterial detection. The samples were formed using silver, zinc oxide, rGO and polyethylene glycol (PEG). GO was formed using a modified Hummers’ method, with graphite powder as the precursor raw material. Then, 2.5 g of agar was put separately into three beakers, each having 100 mL of distilled water. The beakers were heated for one hour at 90 °C at 1000 rpm on a heater cum magnetic stirrer. The beakers were heated for a further 45 min. Each beaker had 0.75 gm of glycerol as a plasticizer, and the beakers were heated until the solutions turned into a semi-sticky liquid. Afterwards, they were heated for 4 h at 35 °C in an air oven. After the chemical modification of the samples, the pH was adjusted to around 8. Finally, the samples were centrifuged and dried in an oven at 60 °C. The coated layers were taken from the dried plates and kept in sealed plastic bags for future analyses. The extensibility and tensile strength of the agar-Ag/ZnO/rGO/PEG (AZGP) films were the key parameters in determining how well they could maintain their integrity in the presence of environmental stress. The mechanical parameters of the films at different thicknesses were between 74.54 and 82.48 m, with or without AZGP2. The results showed that when the AZGP2 concentration in the films increased, the tensile strength increased, but the percentage of elongation at break (EAB) declined. The number of visible colonies that developed on film surfaces during different time intervals was used to assess the antibacterial activity of agar and agar-AZGP films. Even after retaining them for 90 days, the agar film containing 75 mg of AZGP showed no signs of colony formation, whereas the AR25AZGP2 film sample, which contained 25 mg of AZGP, showed a shelf-life of more than 75 days.

Huang et al. [75] explained the research conducted on the use of graphene quantum dots (GQDs) and peroxidase to develop highly sensitive sensors to detect bisphenol A (BPA) in food. The working principle was based on the oxidation of BPA and quenching the fluorescence of GQDs. The GQDs were synthesized with citric acid and sodium hydroxide as processed compounds. The pH of the BPA solutions was adjusted, and a luminescence spectrometer was used to complete the detection process. The detection process was carried out by cutting the sensors into 5 mm × 5 mm dimensions and heating the aqueous BPA solutions to 90 °C for a couple of hours. Real-time testing was carried out on six food samples of different materials. The interference observed between the common ions and amino acids was very little. While the fluorescence intensity increased proportionally with the BPA concentration, these sensors’ operating range and limit of detection (LOD) were 1–1000 mM and 0.4 nM, respectively. The average recovery rates for the real samples ranged between 95.2% and 108.3%, and the relative standard deviation was lower than 3.9%.

Another study explaining the use of graphene was highlighted in [76], where colorimetric and laser desorption–ionization mass spectrometry (LDI–MS) sensors were formed using porous poly (lactic) acid (PLA) and GO. These sensors were used to detect the presence of amines in pork samples. The dual detection process was utilized for screening and quantitatively determining the biogenic amines. The porosity of the PLA films was enhanced using calcium carbonate nanoparticles. Similar to the previous example, the response of these sensors also increased with the corresponding increase in the analyte concentration. The PLA-based colorimetric sensors were fabricated using PLA pallets treated with various synthesis methods such as ultrasonication, curing, soaking and drying methods. The GO-coated filter papers were developed by initially forming GO dispersions under the ultrasonication process. These dispersions were then used to coat the filter with the bath sonication process at a temperature of 120 °C for 20 min. The linear ranges for putrescine and cadaverine biogenic amines were 2–10 mg/mL and 0.1–5 mg/mL, respectively. When LDI–MS was used as substrates over the GO-coated paper, the LODs of putrescine and cadaverine were 0.07 pM and 0.02 pM, respectively.

Another study that highlighted the use of graphene for food-related devices was in the research carried out by Lin et al. [77]. Graphene field-effect transistors (G-FETs) were developed to detect indole, which is a bacterial metabolic volatile molecule. The G-FETs were fabricated in two different ways, with each structure varied in the form of gate structures. The varied structures ensured that the experimentation with indole molecules were conducted in the air and liquid phases. The prototypes were developed on silicon dioxide (SiO_2_) substrates having a thickness of 300 nm. The electrodes were developed using chromium and gold, with 15 nm and 90 nm thicknesses, respectively. The final part included the formation of a monolayer of graphene using a chemical vapor deposition (CVD) process. The repeatability of these G-FETs was achieved using a photolithography process to form samples of 100 × 100 µm. The patterning of graphene was carried out using a PMMA-assisted transfer process. This was executed via chemical treatment, spin-coating, curing and UV-light treatment. The interaction between graphene and the volatile molecules occurred due to the latter’s absorption on the graphene surface via π-π stacking. The sensors could detect indole for a concentration ranging between 10 ppb and 250 ppb with an LOD of 10 ppb.

**Figure 3:**
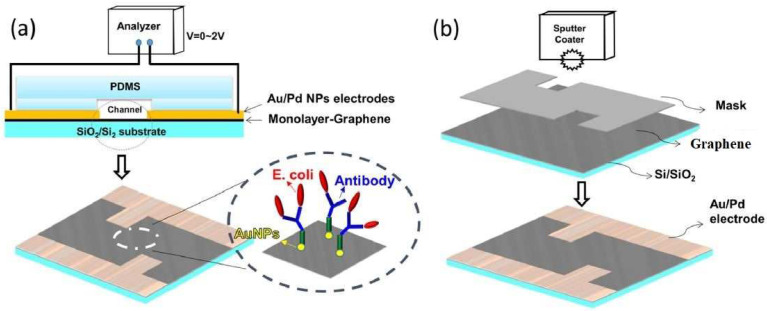
(**a**) Schematic diagram of the structure of the PDMS microchannels. (**b**) The coating of the two sides of the electrodes uses a mask over the upper surface of the graphene [78]. Monolayer graphene chemiresistive biosensor for rapid bacteria detection in a microchannel.

Another study that involved graphene to develop sensors for detecting microorganisms in food is reference [78]. These sensors were chemoresistive in nature, and were formed using monolayer graphene. Some of the advantages of these sensors include their low cost, easy fabrication, high sensitivity and portability. These biosensors were used for bacterial detection purposes, in order to avoid food poisoning issues. The dual conjugation of streptavidin and biotin was achieved to provide immobilization on the graphene surface. The sensors were used to detect *Escherichia coli* (*E. Coli*) through the stemming of anti-*E. Coli* antibody-coated sensing surfaces. Figure 3 [78] shows the schematic diagram of the detection of *E. Coli* in food poisoning, carried out using synthesized anti-*E. Coli*, followed by using it as a coating on the selective layer on the sensing area of the prototypes.

Antibody-coated graphene sensors were used for detection purposes, with the substrate being coated on the two-electrode assembly with a gold/palladium alloy target. Electrical measurement of the resistance values was conducted with respect to the bacterial concentration. This was followed by creating a polydimethylsiloxane (PDMS) microchannel using soft lithography, maintaining the dimensions at 800 µm × 8 mm × 1 mm, perfectly suitable for low-cost technological applications. After rinsing the PDMS microchannels with deionized (DI) water, the sample was incubated with 15 μL of streptavidin, followed by washing it with buffer and DI water and treating it with biotinylated anti-*E. Coli* antibodies. The sample was tested via injection-and-stop and constant injection modes, and showed a resistance change when *E. Coli* binded to the electrode surface. It was seen that the resistance values increased with the bacterial concentration, with the sensors obtaining an LOD of 12 cfu/mL. The change in resistance can be inferred from the formation of a kinetic barrier for the electron transfer process when the bacteria-antibody conjugation process took place on the surface of the graphene film. This led to intercellular bioactivity on the sensing surface and, in turn, polarizing of the charges on the cell’s surface. As a result, there was an increase in the formation of electron clouds due to the steric hindrance, resulting in a change in the carrier hole density. The resistance study was carried out from 0 to 50 min with increments of 5 min, and showed a logarithmic increase in resistance with an *E. Coli* concentration that was enhanced from 2.4 × 5 to 2.4 × 56 cfu mL^−1^.

**Figure 4:**
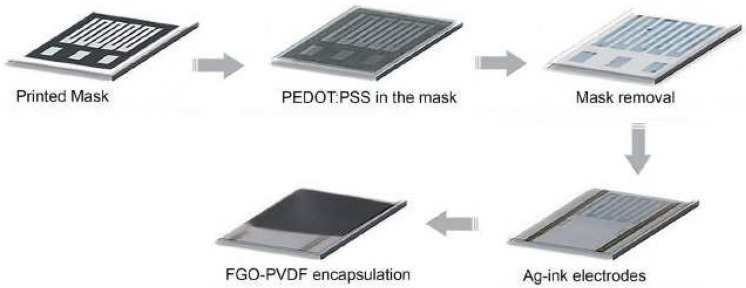
Schematic diagram of the fabrication steps of FGO–PVDF-encapsulated PEDOT: PSS thermistors [79]. Proving the robustness of a PEDOT:PSS-based thermistor via functionalized graphene oxide–poly (vinylidene fluoride) composite encapsulation for food logistics.

Maskey et al. [79] synthesized a functionalized GO–polyvinylidene fluoride (FGO–PVDF) composite to demonstrate the dependability of PEDOT:PSS thermistors in high-humidity conditions. Figure 4 [79] depicts the schematic diagram of the fabrication process of these GO-based sensors. After printing the PEDOT:PSS on PET film and drying it in the oven at 125 °C for 15 min, washing with toluene was performed, followed by solvent evaporation in the oven. The FGO–PVDF composite was prepared using Hummers’ method, where the resulting GO was stirred in dimethylformamide (DMF). Then, phenyl isocyanate was mixed in dichloromethane (DCM), forming amide and carbamate ester-functionalized graphite oxide powder. Then, 1 g of PVDF was dissolved in FGO dispersion in DMF, followed by stirring (12 h) and sonication. The PEDOT:PSS thermistor was painted with Ag ink, followed by encapsulation under inert conditions via drop casting and slow evaporation of the excess solvent at 120 °C. The thermistor was characterized with microscopic techniques such as field-emission scanning electron and atomic force techniques, and showed excellent linearity and stable performance. The resolution was high, with a value of 1272.57 U per °C. Integration of the thermistor was performed into a roll-to-roll (R-2-R) gravure-printed NFC antenna for wireless monitoring of the time–temperature history in the detection of microorganisms, and as a foundation of future smart logistics, which can be considered to be part of the fourth industrial revolution.

Based on the reviewed examples and some other studies, Table 1 shows a comparative study of the performance of the above-mentioned graphene sensors that have been developed and used as sensing prototypes. It can be seen that different physicochemical forms of graphene have been considered to form prototypes due to differences in their physical dimensions, attached functional groups and their abilities to form covalent bonds with target analytes. As mentioned in the table, a wide range of microorganisms, including bacteria, fungi and others, have been detected using these composite and non-composite sensors based on the electrochemical ionic reaction between the electron clouds of the graphene-based active area of the sensors and the functional groups of the target analyte.

## 3. Conclusions and Future Research

This paper presented a substantial review of the fabrication of graphene-based sensors, and their utilization as electrochemical sensors. These prototypes were used to detect different chemicals in food. The graphene sensors were fabricated in composite and non-composite forms. The former involves the inclusion of specific nanomaterials and polymers to improve the sensors’ overall sensitivity and mechanical integrity. Apart from detecting the target analyte, some other advantages of these sensors include their wide operating range, low LOD and biocompatible nature. Microorganisms have been detected in very small amounts due to the excellent morphological characteristics of these sensors, such as their high surface area and superior carrier mobility of graphene. One of the requirements of these sensors would be to detect multiple analytes with equal efficiencies. Different polymers, such as PDMS, poly (lactic) acid and others, have been considered to form composites and substrates due to their excellent capability of forming interfacial bonding with graphene allotropes. The sizes of the sensors also vary based on certain factors such as the size of the target analyte, the working mechanism of the prototypes, and the alignment of graphene in the sensing area.

While a lot of research has been carried out on developing graphene-based sensors to detect various bacterial samples, some research areas are still ongoing. The production of some of these sensors is on a limited scale and are being tested in controlled environments; their ubiquitous utilization in real-time scenarios would help to minimize food wastage to a great extent. Sensors being tested for a single target should opt for more microorganisms that can affect food. This is because when the food samples are transferred in real-time scenarios between the storage unit and superstores, there is a high probability for the growth of microorganisms over a period. The types of wireless communicational protocols associated with graphene-based sensing devices should be increased and optimized on the bases of the maximum and minimum transmission rates, bandwidth and network size. The types of food samples that can be kept safe and edible in different airtight containers integrated with these graphene-based sensors should also be increased to decrease overall food wastage. The temperature and humidity specifications for the sensors should be further optimized to determine the correlation between the detected microorganisms and available food storage facilities. Implementing some of these propositions effectively will benefit mankind regarding production pressure and food hunger.

## Figures and Tables

**Table 1:** Comparative performances of the graphene-based sensors used to detect different microorganisms in food.

Materials	Target Analyte	Detection Technique	Analytical Parameters	Application to Real Samples	Ref.
Graphene	*E. coli*	Field-effect transistors	Size: 100 × 100 µm Operating range: 10–250 ppb LOD: 10 ppb	Milk, meats and seafood	[77]
Graphene, PDMS	*E. coli*	Chemisensitive measurement	Size: 800 µm × 8 mm × 1 mm Operating range: 0–12 cfu/mL LOD: 12 cfu/mL	Meat, milk	[78]
rGO, poly (lactic) acid	*E. coli*	Resistance measurement	Extensive and tortuous diffusion: 1450 times the rGO barrier Operating range: 0–87.6%	Oil and potato chips	[71]
Chitosan, Graphene oxide	*E. coli* (Gram-negative) and *B. subtillis* (Gram-positve)	Differential scanning calorimetry	Tensile strength: 6471.6 ± 1775.5 MPa Operating range: 22.83% (Gram-positive) and 54.93% (Gram-negative)	Food packaging, water treatment	[72]
Polyhydroxybutyrate, graphene nanoplatelets	Polyhydroxybutyrate	Differential thermogravimetric analysis	High melting point, high thermal stability, high tensile strength	Potato chips and milk	[73]
Graphene oxide, gold nanoaparticles	Patulin	Cyclic voltammetry and differential pulse voltammetry	Response time: <1 min LOD: 5 µg/L	Fruits, grains, cheese	[80]
Reduced graphene oxide, tin oxide	Patulin	High-performance liquid chromatography	High recovery rate (74.33 ± 0.70 to 99.26 ± 0.70%) Linear range: 50–600 mM LOD: 0.6635 mM	Apple juice	[81]
Graphene nanoflakes	Citrinin	Molecular Imprinting polymer	High reproducibility (RSD < 5.7%) High accuracy (85.4–111.4%) and recovery LOD: 5 µg/L	Rice, blueberry, corn, wheat, germ, rice starch	[82]
Graphene quantum dots	Yersinia enterocolitica	Immunoassay	High sensitivity and high specificity LOD: 5 cfu/mL	Milk	[83]
Laser induced graphene	Salmonella enterica	Cyclic voltammetry and impedance spectroscopy	Wide linear range (25–10^5^ cfu/mL) LOD: 13 ± 7 cfu/mL	Chicken broth	[84]

## Data Availability

Not applicable.
